# Chinese herbal medicine for patients living with HIV in Guangxi province, China: an analysis of two registries

**DOI:** 10.1038/s41598-019-53725-x

**Published:** 2019-11-25

**Authors:** Jin Sun, Feng Jiang, Bin Wen, Zhen-wei Liu, Mei Han, Nicola Robinson, Nuala McGrath, Yu-tong Fei, Ying Zhang, Jing Li, Wen-yuan Li, Xin Deng, Jian-Ping Liu

**Affiliations:** 1 Centre for Evidence-Based Chinese Medicine, Beijing University of Chinese Medicine, Beijing, 100029 China; 2 Nanjing University of Chinese Medicine, Nanjing, 210023 China; 3 AIDS Centre, Ruikang Hospital, Guangxi University of Traditional Chinese Medicine, Nanning, Guangxi Zhuang Autonomous Region China; 4 School of Health and Social Care, London South Bank University, London, UK; 5 Academic Unit of Primary Care and Population Sciences and Department of Social Statistics and Demography, University of Southampton, Southampton, UK; 6 Research Department of Epidemiology & Public Health, University College London, London, UK; 7 The National Research Centre in Complementary and Alternative Medicine (NAFKAM), Department of Community Medicine, Faculty of Health Science, UiT, the Arctic University of Norway, 9037 Tromsø, Norway

**Keywords:** HIV infections, Epidemiology

## Abstract

Anti-Retroviral Therapy (ART) is the recommended first line therapy for patients with HIV. Since 2004, Chinese government has provided free Chinese herbal medicine (CHM) for Chinese HIV/AIDS patients. Data of living patients with HIV from the NFTCMP database and Center for Disease Control (CDC) database during 2003–2016 in Guangxi province was obtained and compared. Patients were divided into 3 groups according to their recorded treatment regimens. A total of 2954 patients with their treatment recorded in the two databases were included for analysis, their median age was 46 years (IQR = 36–59), and 64.63% were male. CHM regimens users had baseline CD4 cell counts (380.11 ± 240.59 cell/μL), approximately 100 cell/μL significantly higher than patients receiving CHM combined with ART regimens or only ART regimens. There was no significant difference in mortality among groups. All three regimens improved patients’ CD4 cell counts. Compared to the sharp improvement in ART group during the first 6 months, CD4 cell counts of patients in CHM group and CHM combined with ART group showed a smooth and steady rise. CD4 cell counts of the combined group remained much lower than ART group in the first 3 years, but overtook ART group in the fourth year.

## Introduction

Human immunodeficiency virus (HIV) is a retrovirus that can destroy or impair the function of the immune system and which can later progress to Acquired Immune Deficiency Syndrome (AIDS), the most advanced stage of HIV infection. HIV/AIDS remains one of the most important public health problems in the world, particularly in low-income and middle-income countries. According to the World Health Organization (WHO) report^1^, at the end of 2015, there were 36.7 million [34.0 million–39.8 million] people living with HIV, and more than 18 million people were receiving antiretroviral treatment (ART) by mid-2016. Due to treatment, the global annual HIV-related all-cause mortality has continually decreased in recent years. By 2015 the number had reduced to 1.1 million [940 000–1.3 million]. According to the recent report of China Centers for Disease Control (CDC), on October 31, 2017, China’s population of people living with HIV was about 750,000^2^. HIV/AIDS remains one of the most important public health problems in China and no longer restricted to blood donors and drug users but increasingly spreading through the general population due to unsafe sex, especially among men who have had sex with men^3–5^.

Chinese herbal medicine (CHM) was firstly used to treat patients living with HIV in 1987 when Chinese medicine practitioners from China provided medical assistance in Tanzania, Africa^6^. In China, there are three main strategies for treating HIV, which include ART alone, CHM alone, and CHM combined with ART. A pilot project, the National Free CHM HIV/AIDS Treatment Program (NFCHMP), was initiated in 2004 and expanded rapidly. NFCHMP was supported by the State Administration of Traditional Chinese Medicine (SATCHM) and the Ministry of Finance, and the program has provided tens of thousands of HIV/AIDS patients with free Chinese medicine treatment.

A double-blind randomized controlled trial^7^ found CHM could significantly improve CD4 cell counts and symptoms (anorexia, diarrhea, fatigue, nausea, and emesis). In Henan province, a retrospective cohort study based on the database of NFCHMP observed CHM could decrease the disease progression, reduce the mortality of PLHIV, and improve life expectancy. However, the major limitation was that they chose the world mortality rate over the same period as a comparison^8^. A 7-year observational study suggested long-term use of CHM could maintain or slow the pace of CD4 cell counts declining. However, this study did not address bias, potential confounders and the possibility that results may have occurred by chance^9^. CHM has also been reported to relieve HIV/AIDS patients’ clinical symptoms (e.g., fatigue, pain, sleep disturbance, shortness of breath, coughing) and significantly decrease opportunistic infections in before and after studies^10–12^. Although there is some evidence to indicate there was an observable effect of CHM therapy on HIV, most studies had a small sample size and did not address confounding factors. To confirm the effect and safety of CHM, analysis of more longitudinal data is required to analyze the characteristics of the patients who receive CHM therapy, and statistically adjust for confounding factors. Obtaining longitudinal data is expensive, time-consuming, and the data is difficult to analyze, the advantage of longitudinal data is that the individual development of a certain outcome variable can be studied over time. However, when evaluating the effect of CHM for HIV/AIDS, most studies have ignored individual disease progression. With the development of statistical techniques, such as generalized estimating equations (GEE) and random coefficient analysis, it has become possible to analyze longitudinal relationships using all available longitudinal data, without summarizing the longitudinal development of each subject into one value^13^.

Guangxi, a province located in southwestern China that borders Myanmar, has the second highest HIV prevalence in the whole country due to its specific geographical location and cultural environment^14^. Therefore, this study aimed to utilize and explore longitudinal data from the registries established by SATCHM in Guangxi province to analyze the effect of CHM on patients with HIV.

## Methods

### Data sources

The Ethics Committee of Beijing University of Chinese medicine granted ethical approval for analyzing data from two registries (BZYYYDX-LL20160215). This study complied with the Declaration of Helsinki and Good Clinical Practice Guidelines in China. All participants provided their written informed consent and allowed their medical data to be used for researches. To protect participants’ privacy, we extracted all available data without names and other identifiers on HIV patients from two registries (CHM registry and CDC registry) in Ruikang Hospital, which is located in the Guangxi province of southern China. We excluded 29 records that only had an ID number and had no other information.

CHM database collected data HIV/AIDS patients who participated in the NFCHMP and received CHM therapy. In this registered database, all participants were identified from 16 clinical centers across 14 cities. The CDC database in Nanning, the capital of Guangxi province, was also included to compare the effectiveness of CHM therapy and ART therapy. All patients in the two registries had been diagnosed with HIV infection via virus detection.

### Procedure

In the 16 NFCHMP clinical centers, the patients diagnosed with HIV infection would have been informed that NFCHMP could provide free CHM. Patients who had a previous record of severe cerebrovascular disease, severe kidney and hematopoietic system disease, and mental disease were excluded. The NFCHMP participants would have been registered on the CHM registry and would have completed the pre-CHM treatment examination, which we defined as a baseline.

Participants were provided with medication and assessed every three months via an interview and laboratory testing. Doctors identified the CHM therapy according to the infection stage of patients and used the Clinical practice proposal of Traditional Chinese medicine treatment for patients living with HIV/AIDS issued by SATCM. CHM therapy could include Chinese patent medicines such as Tangcao tablets, Shenling Fuzheng capsules, Qingdu capsules, Aifukang capsules, as well as other herbal recipes, depending on patients’ symptoms and according to TCM hospital clinical practice. Whether to take a combined treatment of CHM and ART were decided based on patients’ wishes and symptoms.

According to the CHM registry, all patients took CHM. To compare the effectiveness of CHM with ART, we also accessed the CDC registry and extracted the data of HIV patients who took ART alone in Ruikang hospital.

### Outcome measures

Demographic characteristics included participants’ gender and age were recorded in two registries. CHM registry recorded more demographic information, including marital status, educational levels, and possible route of infection. Mortality rate, CD4, CD8 cell counts, and white blood cell (WBC) counts were extracted to evaluate the effectiveness. We used the abnormal rate of liver and kidney function to evaluate safety. Alanine transaminase (ALT) and aspartate aminotransferase (AST) were used to evaluate liver function. Blood urea nitrogen (BUN) and creatinine (Cr) were used to evaluate kidney function.

### Statistics

The data analyses were performed using SAS version 9.4. We divided participants into different groups by treatment. At baseline, descriptive statistics were used to examine the demographic and clinical characteristics of all participants and in different groups. Continuous variables were summarized as means, standard deviations, median, interquartile range (IQR), range (minimum value to maximum). For categorical variables, the number and percentages of patients in each category were calculated and the proportions of participants who had dropped out, and the meantime of drop-out.

This study provided longitudinal data, in which the outcome variables were repeatedly measured. We used analysis of covariance (ANCOVA) and GEE statistical methods to adjust confounding factors^13^.

Our first approach was an analysis of changes in outcomes between two points in time. Differences between groups were tested for statistical significance using chi-square and Wilcoxon-Mann-Whitney tests for categorical and continuous variables, respectively. Besides, differences between groups were estimated using a one-way analysis of variance (ANOVA) followed by the Student-Newman-Keuls (SNK) test for all pairwise comparisons. Results were considered statistically significant at 0.05 p-value.

ANCOVA used to adjust confounding factors (gender, age, CD4 cell counts at baseline), furthermore, we used GEE to investigate the relationship between the outcome variable CD4 cell counts and the predictor variables (gender, age, group and time), and in the model used in this study, we assumed a linear relationship with time.

### Ethical approval and informed consent

The Ethics Committee of Beijing University of Chinese medicine granted ethical approval for the study.

## Results

### Demographic characteristics

Two thousand nine hundred fifty-four individuals recorded in two registries were extracted for analyses. Of these, 1374 individuals used CHM alone, 1198 individuals used CHM combined with ART, and 382 individuals used ART alone. Participants registered on the databases between 2003 and 2016, the highest proportion (25.84%) were in 2013.

One hundred ten patients dropped out from CHM alone group with the mean drop-out time of 9.11 months. Reasons for dropping out were migrating from the area for work (n = 11), unwilling to take medications (n = 24), patients voluntarily withdrawing from the project (n = 39), travel issues (n = 4), other reasons including: lost touch, compulsory detoxification, being put in prison (n = 32).

Sixty-seven patients dropped out from CHM combined with ART group, and the meantime of drop-out was 17.78 months. They dropped out for the following reasons: migrating from the area for work (n = 3), unwilling to take medications (n = 21), patients voluntarily withdrawing from the project (n = 23), travel issues (n = 4), other reasons including compulsory detoxification and lost-to-follow-up (n = 16).

Of the participants who used CHM therapy and provided demographic characteristics, most participants were married (64.07%), 18.00% were single, 8.10% were divorced, and it is noted that 9.83% were widowed. Further analysis found that, for female patients, the proportion of the widowed (16.86%) was much higher than that of the single (7.58%). On the contrary, for males, the proportion of those who were single (21.24%) was much higher than that of widowed (8.75%). Most participants (40.45%) only received junior middle school education, and few (4.72%) had college or further college education. Most participants (84.38%) considered their possible HIV-infected route was sexual behavior, and drug taking (10.79%) was the second leading possible infection route (Table 1). Individuals used CHM combined with ART had similar but slightly different demographic characteristics. The demographic characteristics of the CHM group and CHM combined with ART group could be compared, but the CDC registry (individuals used ART alone) did not record information on the potential infection route.

**Table 1:** Baseline demographic characteristics of the CHM database stratified by therapy.

Characteristic	ALL (n = 2527)	CHM (n = 1333)	CHM combined with ART (n = 1194)	P value
**Marital status— no. (%)**				
Single	414 (16.38%)	240 (18.00%)	174 (14.57%)	<0.01
Married	1640 (64.9%)	854 (64.07%)	786 (65.83%)	
Divorced	180 (7.12%)	108 (8.10%)	72 (6.03%)	
widowed	293 (11.59%)	131 (9.83%)	162 (13.57%)	
**Educational levels— no. (%)**				
College graduate	113 (4.46%)	63 (4.72%)	50 (4.18%)	<0.01
Senior high school	276 (10.9%)	135 (10.11%)	141 (11.79%)	
Junior middle school	1060 (41.88%)	540 (40.45%)	520 (43.48%)	
Elementary school	887 (35.05%)	467 (34.98%)	420 (35.12%)	
Unschooled	40 (1.58%)	26 (1.95%)	14 (1.17%)	
Preschool	155 (6.12%)	104 (7.79%)	51 (4.26%)	
**possible HIV-infected route**				
Blood transfusion	8 (0.31%)	5 (0.37%)	3 (0.25%)	<0.01
Drug taking	294 (11.57%)	145 (10.79%)	149 (12.44%)	
Sexual behavior	2172 (85.44%)	1134 (84.38%)	1038 (86.64%)	
Mother-to-child transmission	12 (0.47%)	5 (0.37%)	7 (0.58%)	
Paid blood donation	7 (0.28%)	2 (0.15%)	5 (0.42%)	
Unclear	130 (5.11%)	83 (6.18%)	47 (3.92%)	

After data filtering, we analyzed 2637 patients with effective or safety information. The average age of the whole sample was 47.35 ± 14.24, the median age was 45 years (IQR = 36–59), and 65.25% were male (Table 2).

**Table 2:** Baseline demographic and clinical characteristics of the whole cohort stratified by therapy.

Index	ALL (n = 2637)	CHM (n = 1180)	CHM combined ART (n = 1126)	ART (n = 331)	P value
**Sex—no. (%)**					
n	2486	1125	1053	343	<0.01
Male	1622 (65.25%)	694 (63.32%)	716 (68.06%)	212 (62.72%)	
Female	864 (34.75%)	402 (36.68%)	336 (31.94%)	126 (37.28%)	
**Age–yr**					
n	2473	1099	1053	321	<0.01
Mean ± Std	47.35 ± 14.24	47.41 ± 14.74	47.97 ± 13.00	45.12 ± 16.13	
Median	45	45	46	40	
IQR	36, 59	36, 59	37, 59	33, 57	
Range	17–88	17–88	19–84	20–88	
**Age group — no. (%)**					
n	2473	1099	1053	321	<0.01
<20 yr	6 (0.24%)	5 (0.45%)	1 (0.09%)	0 (0%)	
20–29 yr	215 (8.69%)	114 (10.37%)	52 (4.94%)	49 (15.26%)	
30–39 yr	647 (26.16%)	261 (23.75%)	283 (26.88%)	103 (32.09%)	
40–49 yr	579 (23.41%)	260 (23.66%)	264 (25.07%)	55 (17.13%)	
50–59 yr	428 (17.31%)	193 (17.56%)	193 (18.33%)	42 (13.08%)	
60–69 yr	433 (17.51%)	181 (16.47%)	211 (20.04%)	41 (12.77%)	
> =70 yr	165 (6.67%)	85 (7.73%)	49 (4.65%)	31 (9.66%)	
**CD4— cell/μL**					
n	2520	1124	1053	343	<0.01
Mean ± Std	316.85 ± 199.19	376.80 ± 213.53	271.45 ± 181.54	258.17 ± 133.01	
Median	286	372	237	255	
IQR	177, 424	371	237	166, 348	
Range	1–1538	235.5, 488.5	148, 347	1–773	
**CD4 group — no. (%)**					
n	2521	1125	1053	343	<0.01
<350 cell/μL	1551 (61.52%)	499 (44.36%)	794 (75.40%)	258 (75.22%)	
≥350 cell/μL	970 (38.48%)	626 (55.64%)	259 (24.60%)	85 (24.78%)	
**CD8— cell/μL**					
n	2154	1206	943	3	<0.01
Mean ± Std	956.31 ± 521.66	1057.74 ± 550.32	848.67 ± 466.63	778.33 ± 34.96	
Median	860.5	977.5	758	797	
IQR	586, 1223	682, 1344	530, 1079	738, 800	
Range	0–4531	0–4416	48–4531	738–800	
**Platelets—×10**^**9**^**/L**					
n	2637	1180	1126	331	<0.01
Mean ± Std	224.94 ± 74.87	220.45 ± 76.22	233.75 ± 72.71	216.23 ± 73.80	
Median	219	214	226	209	
IQR	179, 266	171, 259	189, 275	177, 248	
Range	0.26–792	2.9–792	0.26–600	14–593	
**Hemoglobin—(g/L)**					
n	2637	1180	1126	331	0.56

### Effectiveness evaluation

During the study period, there were no statistical differences between the groups in mortality (p = 0.24). Seven patients (0.51%) in the CHM group, eight patients (0.67%) in CHM combined with ART group, and five patients (1.31%) in the ART group, died.

The baseline clinical characteristics of the whole cohort were stratified by therapy (Table 2). At baseline, there were significant differences across the three groups regarding age and sex. However, all three were more likely to contain male patients and patients were mostly 30 to 49 years old. CHM regimens users had better baseline status of CD4 cell counts (376.80 ± 213.53 cell/μL, median 371, IQR = 235.5–488.5 cell/μL), around 100 cell/μL higher than the patients with CHM combined with ART regimens (271.45 ± 181.54, median 237, IQR = 148–347 cell/μL) and ART regimens (258.17 ± 133.01, median 255, IQR = 166–348 cell/μL), and there was no significant difference between CHM combined with ART group and ART group. Participants were categorized according to whether the CD4 cell count was greater than 350 per μL. The CHM group had a significantly higher proportion (55.64%) of participants with higher CD4 counts when compared to CHM combined with ART group (24.60%) and ART group (24.78%).

**Figure 1:**
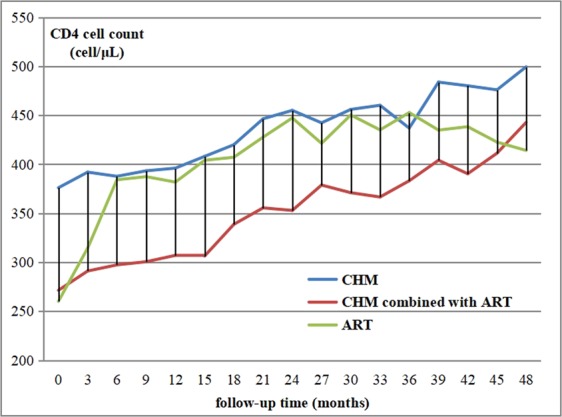
CD4 cell counts curves through 4-years for 3 cohorts stratified by therapy.

Changes in CD4 cell counts of the three cohorts were explored. As most participants had been registered in databases since 2011, four years of data were available. Figure 1 reflected the change curves of CD4 cell counts over a 48-months period for all three groups. All three regimens demonstrated improvements in patients’ CD4 cell counts. Compared to the initial sharp improvement in the ART group, the CD4 cell counts of patients in CHM group and CHM combined with ART group rose steadily and smoothly and respectively peaked at 499.49 ± 199.25 (median 502, IQR = 365–620) and 438.38 ± 200.68 (median 412.5, IQR = 305–535) cell/μL by 48 months. Compared to CHM group and CHM combined with ART group, participants used ART alone had rapidly increasing CD4 cell counts when treatment was initiated and was more likely to slow down their improvement of CD4 cell counts after six months. In the ART group, CD4 cell counts reached a peak at 36 months, before beginning its gradual decline to 414.15 ± 213.68 (median 386.5, IQR = 255–477) cell/μL in 48 months.

We calculated the change of CD4 cell counts from baseline value during the 4-years follow-up period, ANCOVA was used to adjust major confounding factors (age, sex) and compared the differences among groups (Table 3).

**Table 3:** CD4 cell counts of the whole cohort stratified by therapy.

Index	CHM (n = 1180)	CHM combined ART (n = 1126)	ART (n = 331)	Adjusted P value
**change from baseline values at 0.5-yr**				
n	476	436	15	0.26
Mean ± Std	6.47 ± 184.19	31.68 ± 123.94	91.87 ± 121.31	
95% CI	[−10.12, 23.06]	[20.02, 43.35]	[24.69, 159.05]	
Median	0.5	21	42	
IQR	−90, 95	−17, 83	4, 196	
**change from baseline values at 1-yr**				
n	337	344	201	<0.01
Mean ± Std	15.9 ± 192.33	60.35 ± 130.26	130.2 ± 112.41	
95% CI	[−4.71, 36.51]	[46.53, 74.16]	[114.57, 145.84]	
Median	9	43	122	
IQR	−85, 141	−14, 122.5	58, 210	
**change from baseline values at 1.5-yr**				
n	277	206	179	<0.01
Mean ± Std	30.03 ± 175.55	72.39 ± 117.36	158.07 ± 132.15	
95% CI	[9.26, 50.79]	[56.27, 88.51]	[138.58, 177.57]	
Median	32	65	150	
IQR	−88, 160	5, 131	59, 228	
**change from baseline values at 2-yr**				
n	202	147	152	<0.01
Mean ± Std	49.16 ± 198.09	72.12 ± 165.72	198.84 ± 158.79	
95% CI	[21.68, 76.65]	[45.1, 99.13]	[173.39, 224.29]	
Median	57.5	59	176	
IQR	−93, 189	−2, 169	97.5, 267	
**change from baseline values at 2.5-yr**				
n	139	105	123	<0.01
Mean ± Std	70.68 ± 226.16	91.61 ± 166.55	208.91 ± 164.85	
95% CI	[32.75, 108.61]	[59.38, 123.84]	[179.49, 238.33]	
Median	62	85	187	
IQR	−78, 201	3, 180	118, 280	
**change from baseline values at 3-yr**				
n	106	115	82	<0.01
Mean ± Std	49.08 ± 214.89	107.9 ± 176.79	214.91 ± 152.34	
95% CI	[7.69, 90.46]	[75.24, 140.55]	[181.44, 248.39]	
Median	46.5	100	197	
IQR	−76, 164	29, 189	111, 298	
**change from baseline values at 3.5-yr**				
n	63	71	53	0.10
Mean ± Std	93.79 ± 288.11	111.17 ± 161.89	210.75 ± 168.62	
95% CI	[21.24, 166.35]	[72.85, 149.49]	[164.28, 257.23]	
Median	122	110	195	
IQR	−59, 226	35, 207	100, 303	
**change from baseline values at 4-yr**				
n	52	59	31	0.68
Mean ± Std	116.15 ± 229.04	146.63 ± 228.8	231.48 ± 182.6	
95% CI	[52.39, 179.92]	[87, 206.25]	[164.5, 298.46]	
Median	63.5	112	199	
IQR	−62.5, 273	33, 224	112, 335	

Table 4 shows the results of a GEE analysis that was applied to investigate the relationship between the outcome variable CD4 cell counts and the four predictor variables, including gender, age, group, and time. For each of the predictor variables the regression coefficient, the standard error of the coefficient and the corresponding p-value were given. In this model, time was added as a continuous variable coded as [1 to 15], and respectively corresponded to the baseline and the 16 follow-ups during four years.

**Table 4:** Results of a GEE analysis.

Parameter	Estimate	Standard Error	95% Confidence Limits	Z	P
Intercept	466.64	17.18	[432.97, 500.32]	27.16	<0.01
gender	48.73	7.42	[34.20, 63.27]	6.57	<0.01
age	−2.11	0.25	[−2.59, −1.62]	−8.50	<0.01
group	−35.43	5.16	[−45.54, −25.33]	−6.87	<0.01
time	9.43	0.48	[8.48, 10.38]	19.50	<0.01

Gender was significantly related to the development of outcome variable CD4 cell counts. The regression coefficient (β = 48.73, p < 0.01) for gender indicates that there was a greater increase in CD4 counts for females over time. For age, a negative association was found (β = −2.11, p < 0.01). The results also showed that there was a significant linear increase over time in CD4 cell counts (β = 9.43, p < 0.01). The change in CD4 over time was significantly different among groups.

Table 5 shows the laboratory data for each cohort. CD8 cell counts were compared between the three groups by adjusting for age, sex, and baseline values, and there was no significant difference detected during the first 4-year follow-up periods except the 2.5-year.

**Table 5:** Laboratory indexes of the whole cohort stratified by therapy.

Index	CHM (n = 1180)	CHM combined ART (n = 1126)	ART (n = 331)	Adjusted P value
**CD8— cell/μL**				
**0.5-yr**				
n	534	462	15	0.09
Mean ± Std	1026.54 ± 577.33	796.59 ± 465.24	1147.93 ± 631.31	
Median	938	677.5	853	
IQR	639, 1304	481, 1058	756, 1887	
Range	0.54–6548	0.4–3936	332–2303	
**1-yr**				
n	395	368	212	0.80
Mean ± Std	962.73 ± 503.06	796 ± 448.57	902.25 ± 403.66	
Median	878	698.5	838.5	
IQR	595, 1296	465.5, 1010.5	622.5, 1102	
Range	−919–3571	3.92–2710	169–2557	
**1.5-yr**				
n	334	221	187	0.96
Mean ± Std	988.15 ± 494.44	903.42 ± 464.63	897.03 ± 452.45	
Median	892.5	782	817	
IQR	606, 1339	575, 1201	593, 1078	
Range	136–2638	97–2723	150–2819	
**2-yr**				
n	253	161	160	0.86
Mean ± Std	950.55 ± 492.02	881.84 ± 488.51	964.44 ± 478.43	
Median	879	767	869	
IQR	585, 1226	557, 1039	645.5, 1144	
Range	8.4–2840	174–3578	227–2833	
**2.5-yr**				
n	194	119	126	<0.05
Mean ± Std	951.74 ± 498.61	851.51 ± 501.46	975 ± 434.36	
Median	836.5	740	940	
IQR	581, 1224	571, 1090	639, 1191	
Range	0.42–2800	6.07–3990	243–2506	
**3-yr**				
n	146	125	81	0.23
Mean ± Std	944.08 ± 523.42	811.51 ± 457.71	985 ± 493.67	
Median	829	719	855	
IQR	551, 1194	475, 957	640, 1226	
Range	96–2518	115–2705	343–2955	
**3.5-yr**				
n	97	76	53	0.57
Mean ± Std	907.46 ± 438.55	839.16 ± 406.26	916.64 ± 377.64	
Median	782	761	794	
IQR	577, 1110	535.5, 1052.5	642, 1100	
Range	299–2157	218–1976	349–1768	
**4-yr**				
n	87	66	34	0.96
Mean ± Std	914.8 ± 452.13	882.45 ± 609.46	942.21 ± 538.44	
Median	817	731.5	811	
IQR	585, 1204	563, 988	516, 1187	
Range	127–2289	282–4318	332–2828	
**White blood cell—×10**^**9**^**/L**				
**0.5-yr**				
n	785	735	292	<0.01
Mean ± Std	5.64 ± 1.69	5.48 ± 1.74	5.06 ± 1.63	
Median	5.4	5.3	4.91	
IQR	4.55, 6.51	4.37, 6.4	3.96, 6.18	
Range	0.75–16.2	1.67–14.4	1.82–11.44	
**1-yr**				
n	610	619	252	<0.05
Mean ± Std	5.62 ± 1.75	5.46 ± 1.79	5.3 ± 1.61	
Median	5.39	5.15	5.1	
IQR	4.42, 6.48	4.23, 6.4	4.18, 6.23	
Range	1.72–18.7	2.06–15.8	1.45–11.53	
**1.5-yr**				
n	492	357	252	0.51
Mean ± Std	5.69 ± 1.64	6.39 ± 15.78	5.3 ± 1.61	
Median	5.44	5.38	5.1	
IQR	4.43, 6.63	4.39, 6.42	4.18, 6.23	
Range	1.5–12.4	1.89–301	1.45–11.53	
**2-yr**				
n	390	296	199	0.59
Mean ± Std	5.61 ± 1.6	6.49 ± 17.65	5.78 ± 3.56	
Median	5.45	5.32	5.37	
IQR	4.5, 6.5	4.39, 6.35	4.51, 6.47	
Range	2.46–11.8	2.23–308	2.09–50.2	
**2.5-yr**				
n	309	229	178	0.38
Mean ± Std	5.66 ± 1.71	5.69 ± 1.88	5.47 ± 1.53	
Median	5.4	5.44	5.31	
IQR	4.4, 6.71	4.4, 6.59	4.46, 6.3	
Range	2.7–13.07	2–16.66	2.4–10.89	
**3-yr**				
n	219	169	129	0.59
Mean ± Std	5.62 ± 1.57	5.55 ± 1.8	5.56 ± 1.62	
Median	5.47	5.27	5.41	
IQR	4.5, 6.6	4.2, 6.5	4.62, 6.23	
Range	2.33–11.3	2.21–11.9	2.25–11.78	
**3.5-yr**				
n	163	129	84	0.10
Mean ± Std	5.47 ± 1.68	5.28 ± 1.54	5.49 ± 1.6	
Median	5.3	4.94	5.31	
IQR	4.4, 6.2	4.2, 6.08	4.18, 6.32	
Range	2.79–14.68	2.2–10.9	2.59–9.95	
**4-yr**				
n	125	94	57	0.35
Mean ± Std	8.92 ± 36.02	5.97 ± 1.79	5.29 ± 1.44	
Median	5.5	5.57	5.26	
IQR	4.71, 6.3	4.7, 7	4.44, 5.96	
Range	2.48–408	3–12.1	2–9.99	

At baseline, comparing to participants used ART alone (5.16 ± 1.73, median 4.97, IQR = 3.97–6.1 × 109/L), participants in CHM group (5.71 ± 1.85, median 5.4, IQR = 4.55–6.53 × 109/L) and CHM combined with ART group (5.5 ± 2.26, median 5.2, IQR = 4.1–6.54 × 109/L) were more likely to have higher white blood cell (WBC) counts. After one year of treatment, there were no statistical differences in WBC counts among groups, and the mean and median WBC counts were all within normal limits.

### Safety evaluation

**Figure 2:**
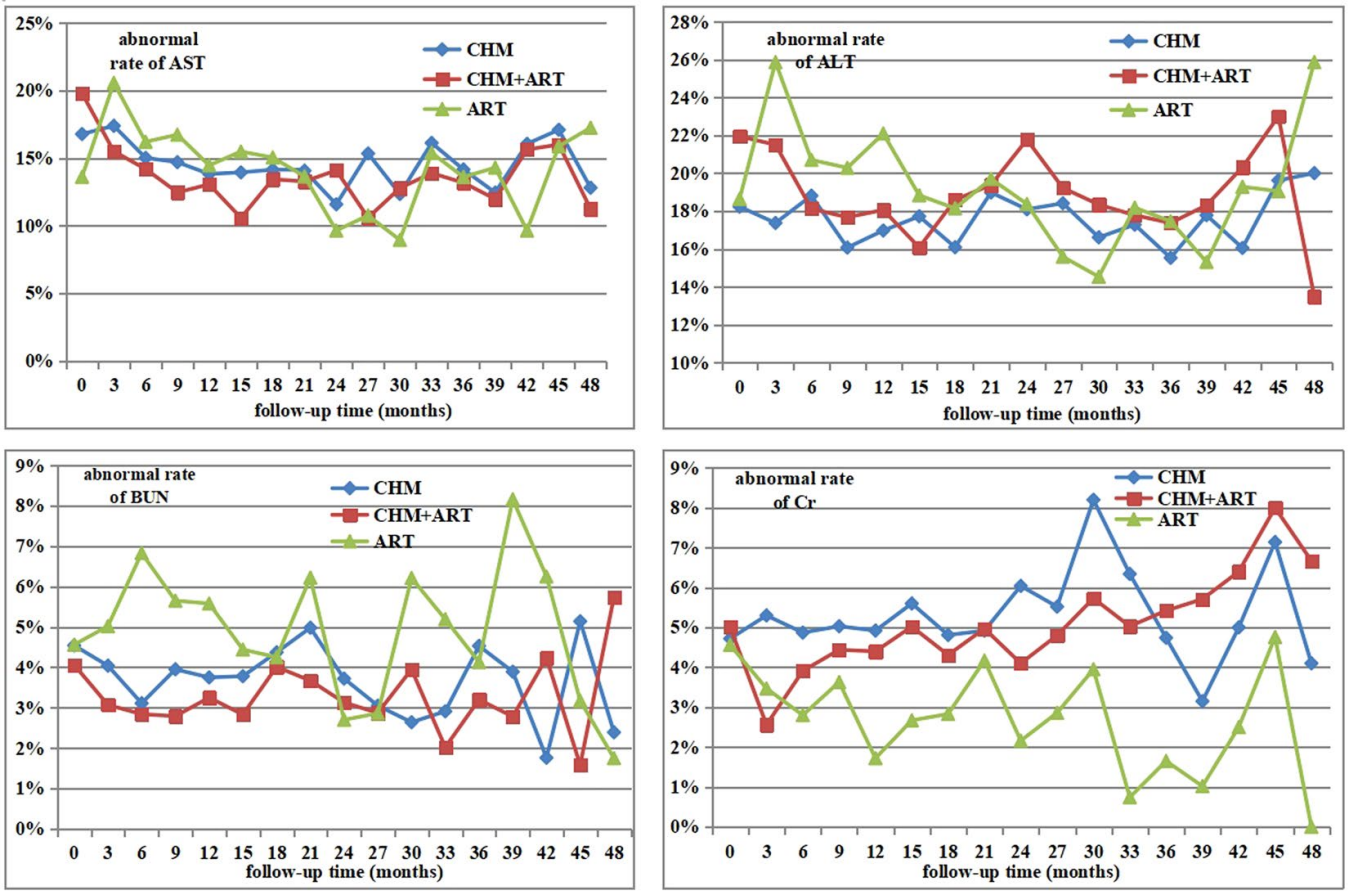
Abnormal rate of liver and kidney function through 4-years for 3 cohorts stratified by therapy.

Figure 2 shows the abnormal rate of liver and kidney function throughout the 4-years for the three groups. For liver function, at baseline, the abnormal rate of AST in CHM combined with ART group (19.79%) was significantly (**χ**^**2**^ = 7.73, P = 0.02) higher than the ART group (13.61%). The abnormal rate of ALT had significant differences among groups. In the first three months of ART treatment, liver function abnormal rate significantly increased and significantly higher than the other two treatments. However, after more than three months of treatment, no significant difference was detected among groups. Regarding kidney function, BUN and Cr were not significantly different at baseline between the groups. For patients taking CHM and CHM combined with ART, and ART, the baseline BUN abnormal rate was 4.54%, 4.06%, and 4.56% respectively. There was no significant difference. During four years of treatment, only at 48 months was a significant difference detected between CHM combined with ART and ART alone. Three groups had similar Cr at baseline. During the treatment, the abnormal rate for the CHM group was significantly higher than for the CHM combined with ART at 3 months. Additionally, at 33 months, CHM had a significantly higher abnormal rate than ART.

## Discussion

This preliminary analysis showed that CHM gradually and continuously improved CD4 cell counts of patients with a relatively better baseline CD4 status. Compared to patients who only took the ART regimen, the patients who combined CHM were more likely to have a steady and continuous growth in CD4 cell counts. This phenomenon indicated there might be some drug interactions between two therapies. We found that for the patients who had relatively higher CD4 cell counts (>350 cell/μL) and did not take ART before, CHM could steadily increase CD4 cell counts and improved immunity levels. Compared to using ART alone, the CD4 cell counts of participants using CHM combined with ART increased slower in the first three years. It may relate to the potential effect of CHM on promoting immune reconstitution and counteracting the adverse side effects of antiviral drugs^15^. A study has suggested that the asymptomatic stage is the optimal time to take CHM, for it could help to maintain and enhance the immune function so as to delay the progression to the AIDS stage. For those patients who have a diagnosis of AIDS, CHM focuses on relieving symptoms of patients having AIDS-related opportunistic infection^16^. There was no statistical difference in the death rate among the three groups, and it may relate to the generally low death rate of patients with HIV.

In China, governments provide patients with free antiviral drug including nucleoside reverse transcriptase inhibitor (NRTI), non-nucleoside reverse transcriptase inhibitor (NNRTI), and protease inhibitor (PI), such as inhibitor didanosine (ddI), lamivudine (3TC), stavudine (d4T), zidovudine (AZT, ZDV), nevirapine (NVP), and indinavir (IDV). Using a combination of d4T, 3TC and NVP are recommended as the first-line treatment for patients who had not received ART. For those patients who had accepted ART before, experts decide whether they need to change by considering their condition and personal willingness^17^.

As a chronic disease, the safety of long-term medication is an important issue that both patients and doctors concerned with. In this study, long-term CHM alone or ART combined regimens seemed safety. After the first three months of treatment, only patients who used ART alone had a significant increase in the abnormal rate of liver function. While patients used CHM alone and CHM combined with ART had no significant change in liver function.

The strength of our study was our use of both traditional statistical methods and GEE to analyze longitudinal data. This study has some limitations. The CHM database was based on the patients who had agreed to take part and who completed the full four years follow up and possibly did not reflect all those taking CHM. There may also have been some selection bias with those who accepted CHM as a treatment or as part of their treatment regimen. This was not a randomized controlled trial, and therefore, potential selection bias of whether or not to consent to the study may have influenced the results.

## Conclusions

Three therapies appeared to improve CD4 cell counts of patients in the first three years. After three years, CHM alone or combined with ART could still improve CD4 cell counts. There was no evidence to suggest CHM could decrease mortality. It is noteworthy that compared to patients who only took ART, patients taking CHM were more likely to have a steady rise in CD4 cell counts.

## Data Availability

The datasets generated during and/or analyzed during the current study are available from the corresponding author on reasonable request.
